# Coronal plane alignment changes do not affect in vivo kinematics for robotically performed total knee arthroplasty

**DOI:** 10.1002/jeo2.70776

**Published:** 2026-05-26

**Authors:** Cham‐Kit Wong, Michael LaCour, Garett Dessinger, Richard D. Komistek, Rex Wang‑Fung Mak, Jonathan Patrick Ng, Gloria Yan‑Ting Lam, Tsz‑Lung Choi, Mingde Cao, Kevin Ki‑Wai Ho, Michael Tim‐Yun Ong, Patrick Shu‐Hang Yung

**Affiliations:** ^1^ Department of Orthopaedics and Traumatology Prince of Wales Hospital Shatin Hong Kong SAR, China; ^2^ Department of Mechanical, Aerospace, and Biomedical Engineering Center for Musculoskeletal Research University of Tennessee, Knoxville Knoxville Tennessee USA; ^3^ Department of Orthopaedics and Traumatology Alice Ho Miu Ling Nethersole Hospital Tai Po Hong Kong SAR, China; ^4^ Department of Orthopaedics and Traumatology The Chinese University of Hong Kong Shatin Hong Kong SAR, China; ^5^ Department of Orthopaedics and Traumatology Chinese University of Hong Kong Medical Centre Shatin Hong Kong SAR, China

**Keywords:** coronal plane alignment, CPAK classification, in vivo kinematics, robotic‐assisted surgery, total knee arthroplasty

## Abstract

**Purpose:**

Total knee arthroplasty (TKA) is effective for alleviating pain and restoring function in knee osteoarthritis patients, yet 10% report dissatisfaction due to persistent pain and functional limitations. Conventionally, mechanically aligned (MA) TKA often fail to replicate native kinematics. Kinematic alignment (KA) aims to replicate pre‐arthritic alignment for better function. The coronal plane alignment of the knee (CPAK) system classifies knees into nine groups. Changes in CPAK may alter load distribution, ligament tension and joint congruence. Conflicting studies exist on the impact of CPAK changes on outcomes. No prior study has used fluoroscopy to examine weight‐bearing kinematics stratified by CPAK changes post‐TKA.

**Methods:**

This retrospective analysis used data from a registry of primary robotic‐assisted TKAs (2019–2023) using NAVIO/CORI (Smith & Nephew) systems, with Journey II BCS implants. Exclusions: prior ipsilateral surgery, complicating conditions or missing radiographs. Procedures used MA or KA per surgeon preference, with standardized protocols. Demographics, operative data, pre/post‐operative Knee Society Scores (KSS/KSFS) at 12 months were collected. CPAK was calculated from radiographs. Patients performed weight‐bearing deep knee bends under fluoroscopy; kinematics were extracted via 3D‐to‐2D registration, assessing flexion, condylar positions and axial rotation. Cohorts: CPAK change (*n* = 33) versus no change (*n* = 9). Student's *t* test compared outcomes (*p* < 0.05).

**Results:**

No differences in maximum flexion (Change: 105 ± 11.9° vs. No Change: 101 ± 11.4°, *p* = 0.42), lateral condyle rollback (−13.1 ± 5.6 mm vs. 10.1 ± 5.2 mm, *p* = 0.15), medial condyle rollback (−5.9 ± 3.1 mm vs. −4.7 ± 2.7 mm, *p* = 0.29) or axial rotation (8.9 ± 5.9° vs. 6.5 ± 4.1°, *p* = 0.27). KSFS improvements (+28 ± 21.1 vs. +17 ± 19.2, *p* = 0.16) and KSS (+38 ± 19.2 vs. +38 ± 25.9, *p* = 0.95) were similar. Post‐operative CPAK 3 was the most frequent (45.2%).

**Conclusion:**

CPAK changes after robotic‐assisted TKA do not affect in vivo kinematics or short‐term outcomes, supporting alignment flexibility with precise techniques.

**Level of Evidence:**

Level IV.

**Abbreviations**
aHKA: arithmetic hip–knee–ankle angle
BCS: bi‐cruciate stabilized
CPAK: coronal plane alignment of the knee
FJS‐12: Forgotten Joint Score
HKA: hip–knee–ankle angle
JLO: joint line obliquity
KA: kinematic alignment
KOOS‐12: Knee Injury and Osteoarthritis Outcome Score
KSFS: Knee Society Function Score
KSS: Knee Society Score
LAP: lateral anterior/posterior position
LDFA: lateral distal femoral angle
MA: mechanically aligned
MAP: medial anterior/posterior position
MPTA: medial proximal tibial angle
PROM: patient‐reported outcome measure
TKA: total knee arthroplasty

## INTRODUCTION

Total knee arthroplasty (TKA) is a highly effective surgical intervention, widely performed to alleviate pain and restore function in patients with symptomatic knee osteoarthritis (OA), significantly enhancing their quality of life [13]. Nevertheless, approximately 10% of patients remain dissatisfied [6], potentially due to the inability of conventional techniques to reproduce native, patient‐specific knee kinematics [3, 7]. This has led to ongoing efforts to optimize alignment strategies in TKA.

Historically, mechanically aligned (MA) TKA has been the gold standard, aiming to position implants perpendicular to the mechanical axis of the femur and tibia to ensure balanced load distribution and promote implant longevity [8]. Although associated with excellent survivorship, MA does not consistently restore native alignment, which often deviates from neutral [4]. Kinematic alignment (KA) aims to replicate pre‐arthritic anatomy and joint line orientation to better restore natural biomechanics [12]. However, comparative studies frequently report minimal differences in patient‐reported outcomes between MA and KA [9, 26].

The coronal plane alignment of the knee (CPAK) classification system provides a standardized framework for categorizing knee alignment pre‐ and post‐operatively [18]. The CPAK system classifies knees into nine distinct groups based on the medial proximal tibial angle (MPTA) and lateral distal femoral angle (LDFA), from which joint line obliquity (JLO) and arithmetic hip–knee–ankle angle (aHKA) are derived. Biomechanically, when post‐operative alignments result in changes in CPAK alignment compared to the preoperative classification, this potentially affects the joint function in three ways. First, when CPAK is altered after TKA, the load distribution across the medial and lateral compartments can be altered [10, 16]. Second, although CPAK is a static 2D measurement, the resulting JLO and collateral ligament tensions establish the baseline biomechanical environment that dictates dynamic 3D kinematics throughout the flexion arc. Ophoff et al. [21] demonstrate that medial and lateral collateral ligament elongation patterns are phenotype‐dependent. CPAK alternation will shift where on the force‐elongation curve each ligament operates. Finally, joint congruence also contributes to the kinematics. The fit between the femoral and tibial articular surfaces is influenced by alignment. Klasan et al. [14] found that KA reduces or has comparable contact pressures on the PE bearing surface by increasing or maintaining the contact area throughout one gait cycle in a validated finite element analysis model. Misalignment can lead to incongruence, altering the knee's ability to move smoothly through its range of motion.

The clinical implications of altering a patient's native joint line and CPAK classification remain a subject of debate. Some studies, such as Agarwal et al. [1], suggest that modifications to the native joint line do not significantly impact post‐operative satisfaction. Similarly, Al‐Abbasi et al. [2] found no notable differences in patient‐reported outcome measures (PROMs) or implant survivorship associated with changes in CPAK phenotype. On the other hand, Konishi et al. [15] reported that alterations in varus/valgus alignment negatively influenced outcomes, as measured by the Knee Injury and Osteoarthritis Outcome Score (KOOS‐12) and Forgotten Joint Score (FJS‐12), highlighting the potential adverse effects of alignment changes. Additionally, a study recently [24] demonstrated that changes in CPAK classification following robotic‐assisted TKA with the Journey II implant system did not adversely affect functional outcomes, suggesting that implant design may mitigate alignment‐related disparities. These conflicting findings underscore the complexity of knee biomechanics and the lack of consensus on the optimal alignment strategy for TKA. This necessitates further investigations into the interplay between alignment, implant design and post‐operative kinematics.

Thus, the aim of this study is to evaluate in vivo, weight‐bearing kinematics in TKA patients with and without CPAK change and to use fluoroscopic analysis [19] to assess the impact of post‐operative CPAK changes compared to preoperative classifications, providing novel insights into the relationship between coronal plane alignment and kinematic outcomes after TKA. To the authors' knowledge, no study has employed fluoroscopy to specifically investigate the weight‐bearing kinematics of TKA patients stratified by CPAK classification or changes in CPAK post‐surgery.

## METHODS

### Study design

This retrospective study analyzed data from a prospective institutional joint registry of patients who underwent primary robotic‐assisted TKA at a tertiary centre between 2019 and 2023, performed by a consistent team of experienced orthopaedic surgeons specializing in arthroplasty. Ethical approval was granted by the local research ethics committee.

### Patient selection

Patients who underwent primary TKA with bi‐cruciate stabilized (BCS) implant using the NAVIO or CORI robotic systems (Smith and Nephew) were included in this study. Exclusion criteria were (1) prior ipsilateral knee surgery, including previous knee arthroplasty or osteotomy, (2) underlying disease or complicating conditions, including previous periarticular fracture, severe fixed flexion contracture > 20°, multi‐ligament instability, bone stock deficiency requiring augmentation and stems, neuromuscular disorder, acute and chronic infection and (3) absence of preoperative or post‐operative long leg radiographs or patients lost to follow‐up.

### Technique

All TKA procedures were performed using either mechanical alignment (MA) or kinematic alignment (KA), based on the surgeon's preference. In the KA group, a classic KA workflow was followed (pre‐resection gap assessment → surgical planning → distal femur resection first → tibial resection), rather than an inverse KA (iKA) technique. All subjects were implanted by a consistent team of experienced arthroplasty surgeons employing the respective robotic platform. Standardized wound closure techniques and post‐operative recovery protocol, including perioperative analgesic and antiemesis measures, were implemented as part of the adult joint reconstruction enhanced recovery after surgery protocol. A standardized physiotherapy rehabilitation protocol for adult joint reconstruction was followed, and patients were discharged when mobility permitted outpatient care.

### Clinical information

Clinical data collected included the patients' demographic data, operation records, preoperative and post‐operative functional scores. Knee Society Function Score (KSFS) and Knee Society Score (KSS) data were collected preoperatively and 12 months post‐operatively, and changes in each score are reported.

### Radiographic measurements

Preoperative and post‐operative CPAK calculations were conducted according to the protocol outlined by MacDessi et al. [18]. The LDFA and MPTA were measured radiographically. LDFA was defined as the angle between the femoral mechanical axis and the tangent to the distal femoral articular surface, while MPTA was the medial angle between the tibial articular marginal line and the mechanical axis from the ankle centre to the tibial spine centre. From these, the preoperative aHKA was calculated as MPTA minus LDFA, and JLO as the sum of MPTA and LDFA.

Subsequently, study participants were divided into two cohorts based on whether their CPAK classification remained the same post‐operatively as it was preoperatively (‘No Change’ cohort) or if the CPAK classification changed post‐operatively (‘Change’ cohort). Correlations between the CPAK classification changes and both clinical outcomes and in vivo 3D kinematics were evaluated.

### Kinematics analysis

To determine weight‐bearing kinematics, all subjects were asked to perform a weight‐bearing deep knee bend activity under fluoroscopic surveillance. Subjects began standing with their knees at full extension (0° of flexion) and weight evenly distributed between their legs. They were then asked to bend down and flex their knees as far as they were able. Patients were instructed to perform the activity in a comfortable manner that allowed for the greatest weight‐bearing flexion. The fluoroscopic video was saved and corrected for distortion. Kinematics were extracted using previously published 3D‐to‐2D image registration techniques [19]. Specifically, 3D models of the implanted components were superimposed atop the 2D fluoroscopic image, and their position was modified until the 3D model silhouette aligned with the fluoroscopic silhouette of the implanted components. This was performed for frames at 30° increments throughout the flexion cycle, and the data were interpolated between frames. While in‐plane errors are <0.65 mm and 1.5°, out‐of‐plane translation errors (medial/lateral) are <1.5 mm, and out‐of‐plane rotation errors (varus/valgus) are <2.0° [17, 20]. Specific kinematic parameters of interest in this study included maximum weight‐bearing flexion, lateral anterior/posterior position (LAP, positive is anterior), medial anterior/posterior position (MAP, positive is anterior) and femorotibial axial rotation (positive is external rotation).

### Statistical analysis

Data are expressed in mean, standard deviation and quartile. Data normality was assessed using the Shapiro–Wilk test prior to applying parametric tests. Changes in CPAK, KSS and KSFS were compared between groups using either the Student's *t* test or the Mann–Whitney *U* test, as appropriate. For all analyses, *p* values of less than 0.05 were considered statistically significant. All statistical analyses were performed using IBM SPSS version 28 (IBM Corp).

## RESULTS

In‐vivo 3D kinematics were determined for 42 subjects, 33 who experienced a change in CPAK classification post‐operatively compared to preoperatively, and 9 who did not experience a CPAK classification change (Figure S1), with the most common transition patterns (CPAK I–III, 13 cases). The Change cohort demonstrated a higher post‐operative TKA JLO, while no statistically significant difference was observed in aHKA (Figure S2).

**Figure 1:**
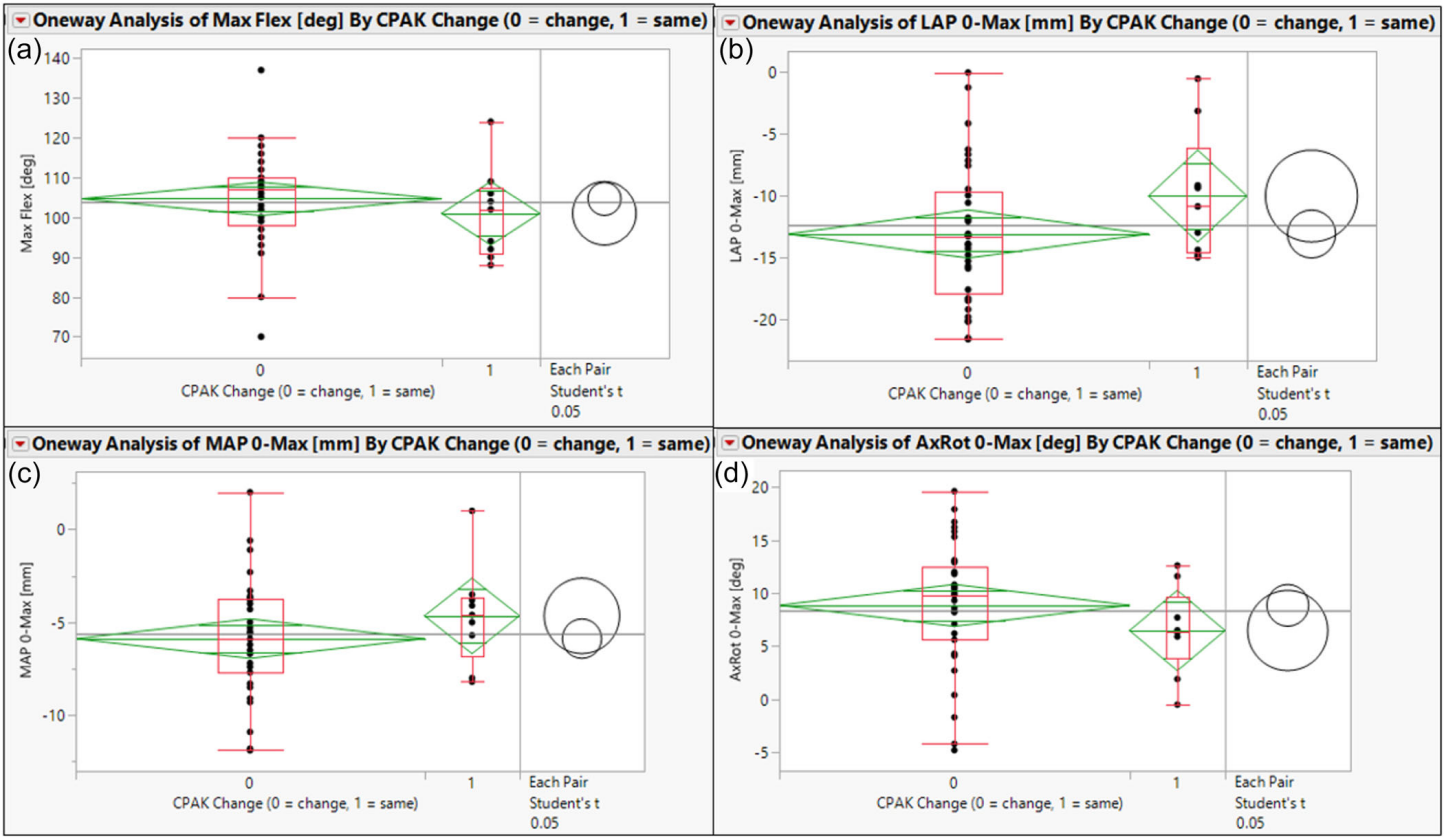
Statistical visualizations of mean, standard deviation, quartile and *t* testing data for Change and No Change comparisons for (a) maximum flexion, (b) lateral condylar translation, (c) medial condylar translation and (d) femorotibial axial rotation.

Maximum weight‐bearing flexion did not differ significantly between cohorts (mean difference: 3.6°, 95% confidece interval [CI]: −5.4° to 12.6°, *p* = 0.42) (Figure 1a, Table 1).

**Table 1:** Comparison of kinematic and clinical outcomes between CPAK change and no‐change cohorts.

Outcome	Change (*n* = 33), Mean ± SD	No Change (n = 9), Mean ± SD	Mean difference (Change − No Change)	95% CI	*p*
Maximum flexion (°)	104.6 ± 11.9	101.0 ± 11.4	3.6	−5.4 to 12.6	0.42
Lateral condyle rollback (mm)	−13.12 ± 5.6	−10.06 ± 5.2	−3.06	−7.24 to 1.12	0.15
Medial condyle rollback (mm)	−5.88 ± 3.1	−4.66 ± 2.7	−1.23	−3.52 to 1.07	0.29
Axial rotation (°)	8.85 ± 5.9	6.48 ± 4.1	2.37	−1.89 to 6.64	0.27
KSFS improvement^a^	27.96 ± 21.1	16.67 ± 19.2	11.30	−4.86 to 27.46	0.16
KSS improvement^a^	37.70 ± 19.2	38.22 ± 25.9	−0.52	−16.90 to 15.86	0.95

Abbreviations: CI, confidence interval; CPAK, coronal plane alignment of the knee; KSFS, Knee Society Function Score; KSS, Knee Society Score; SD, standard deviation.

^a^ Clinical outcome data were available for 36 subjects (*n* = 27 vs. 9).

**Figure 2:**
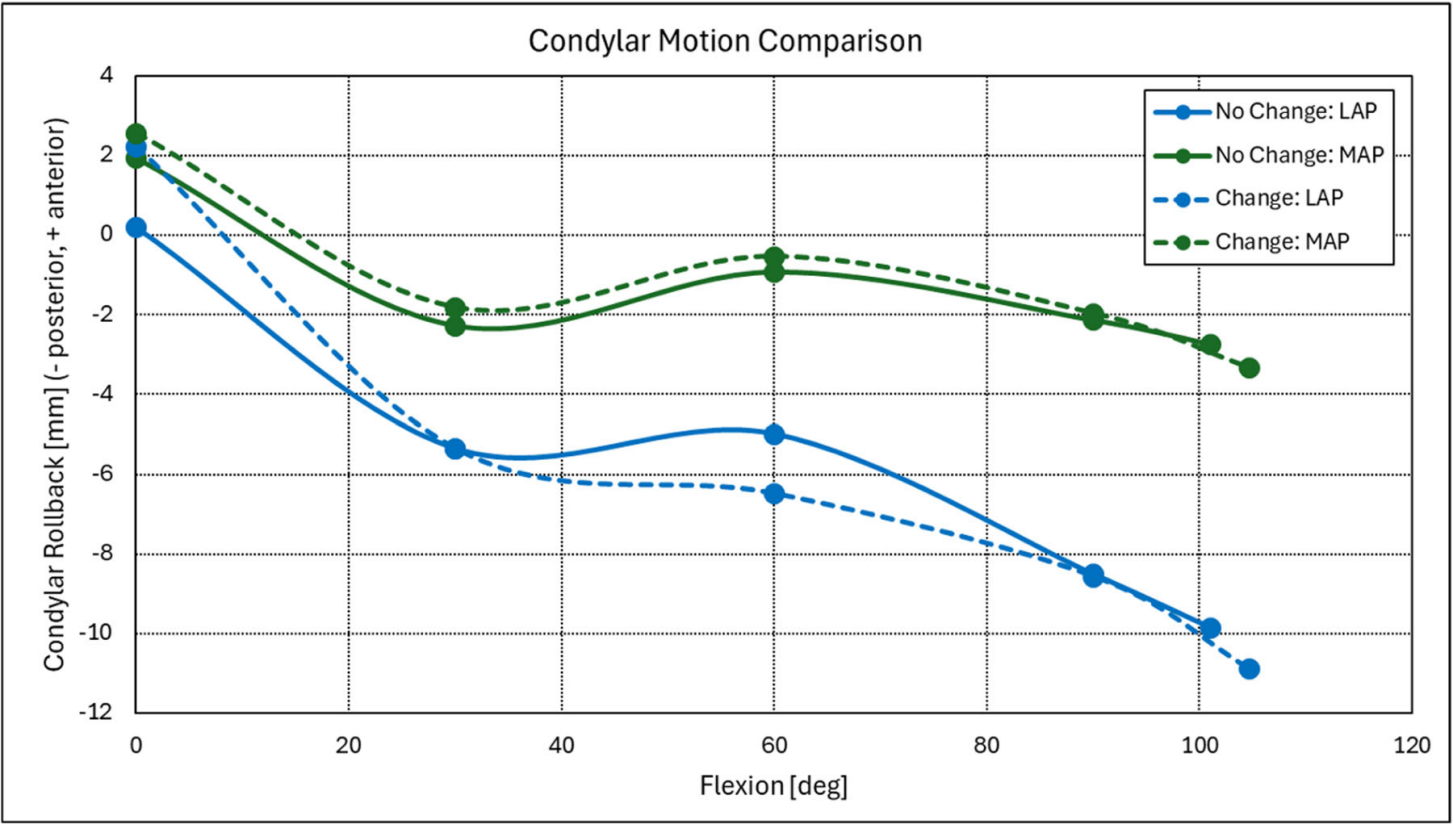
Femorotibial condylar kinematics for both Change and No Change cohorts with increasing flexion during a deep knee bend activity. CPAK, coronal plane alignment of the knee; KSFS, Knee Society Function Score; KSS, Knee Society Score.

Kinematically, there were no differences in total femorotibial condylar motion from full extension to maximum flexion between cohorts (Figures 1b,c and 2). Specifically, Lateral femoral condyle rollback also did not differ significantly between groups (mean difference: −3.06 mm, 95% CI: − 7.24 to 1.12 mm, *p* = 0.15) (Figure 1b, Table 1). Similarly, medial condyle rollback was similar between cohorts (mean difference: −1.23 mm, 95% CI: − 3.52 to 1.07 mm, *p* = 0.29) (Figure 1c, Table 1).

**Figure 3:**
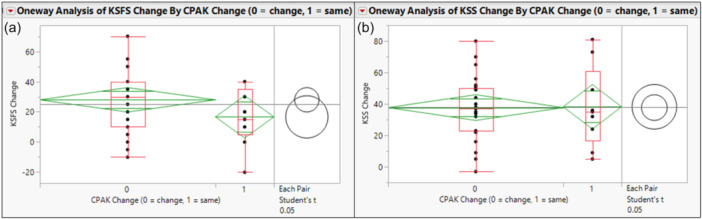
Statistical visualizations of mean, standard deviation, quartile and *t*‐testing data for Change and No Change comparisons for (a) KSFS change from preoperative to 12 months post‐operative and (b) KSS change from preoperative to 12 months post‐operative. KSFS, Knee Society Function Score; KSS, Knee Society Score; LAP, lateral anterior/posterior position; MAP, medial anterior/posterior position.

External femorotibial axial rotation did not differ significantly between cohorts (mean difference: 2.37°, 95% CI: − 1.89° to 6.64°, *p* = 0.27) (Figure 1d, Table 1). Both cohorts demonstrated post‐operative improvements in clinical scores. The magnitude of improvement was comparable between groups for KSFS (mean difference: 11.30, 95% CI: − 4.86 to 27.46, *p* = 0.16) and KSS (mean difference: −0.52, 95% CI: − 16.90 to 15.86, *p* = 0.95) (Figure 3b, Table 1).

Regardless of preoperative classification, CPAK Classification 3 was the most frequent post‐operative phenotype (45.2%), whereas CPAK Classification 8 was not observed post‐operatively (0%) (Table 2).

**Table 2:** Post‐operative CPAK classification frequency, regardless of preoperative classification.

Post‐operative CPAK classification		
CPAK 1	CPAK 2	CPAK 3
6/42 (14.3%)	6/42 (14.3%)	19/42 (45.2%)
CPAK 4	CPAK 5	CPAK 6
1/42 (2.4%)	3/42 (7.1%)	4/42 (9.5%)
CPAK 7	CPAK 8	CPAK 9
1/42 (2.4%)	0/42 (0.0%)	2/42 (4.8%)

Abbreviation: CPAK, coronal plane alignment of the knee.

## DISCUSSION

Although the CPAK classification system is a relatively new classification, understanding the implications that various component alignment techniques have on post‐operative alignment and biomechanics is essential to optimizing post‐operative outcomes. This becomes especially important alongside the rise in patient‐specific alignment techniques such as kinematic alignment, as well as its various restricted versions. While MA would seek to create a post‐operative CPAK classification of V (perpendicular JLO and perpendicular aHKA) for all patients, regardless of preoperative classifications, unrestricted KA would seek to keep the preoperative CPAK classification unchanged, regardless of preoperative deformity. Alternatively, restricted versions of KA, where surgeons may not feel comfortable with a JLO in excess of 3° of varus (or valgus), may seek to maintain aHKA while restricting the JLO, thereby targeting post‐operative classifications of 4, 5 and 6. Unfortunately, there seems to be conflicting data present in the literature, which makes data interpretation and surgical guidelines difficult to solidify.

This study demonstrates that changes in CPAK classification following robotically performed TKA do not significantly affect in vivo kinematics or short‐term clinical outcomes at 12 months. Both cohorts, with and without CPAK changes, exhibited comparable maximum weight‐bearing flexion, condylar motion, and femorotibial axial rotation, as well as similar improvements in KSFS and KSS. Theoretically, altering the native CPAK classification changes joint line obliquity, which shifts the collateral ligaments' operating lengths and could manifest as altered condylar rollback or axial rotation. The lack of observed kinematic differences suggests that the inherent stability of the BCS implant design and the precision of robotic soft‐tissue balancing may override the kinematic consequences of coronal alignment changes.

These findings align with studies suggesting that altering the native joint line does not significantly impact outcomes. Agarwal et al. [1] reported no effect of CPAK changes on patient satisfaction in robotic‐assisted TKA, and Al‐Abbasi et al. [2] found no differences in PROMs or implant survivorship. A recent randomized controlled trial further supports these results, showing comparable PROM improvements regardless of CPAK changes in both conventional and robotic‐assisted TKA [5]. However, Konishi et al. [15] reported that alignment changes negatively affected outcomes, as measured by the KOOS‐12 and FJS‐12. These discrepancies may stem from differences in surgical techniques, implant designs, or patient populations. For instance, our study utilized robotic assistance, which enhances precision in alignment and soft tissue balancing, potentially mitigating the effects of CPAK changes [22].

In this study, most patients ended with post‐operative CPAK alignment classifications of 3. The predominance of post‐operative CPAK 3 is potentially heavily influenced by the Journey II implant design. This built‐in geometry mathematically shifts the derived aHKA toward valgus, acting as a classification artefact rather than a true reflection of surgical valgus alignment. Interestingly, a significant amount of post‐operative CPAK have aHKA in valgus alignment (positive). This is because a large portion of our cohort is using the Journey II system with a 3° in‐built varus in the femoral component. The LDFA is 3° less; by MPTA–LDFA, it is more likely to have a positive aHKA (valgus) while the actual measured HKA is neutral (zero) or even varus (negative). Regardless, there does not appear to be a difference in post‐operative kinematics nor PROM score changes for patients in this study who experienced a change in CPAK classification compared to those who did not. Neither cohort experienced differences in lateral and medial condylar motion or overall femorotibial stability. Future analyses should also incorporate the functional knee phenotypes described by Hirschmann et al., which provide a higher‐resolution framework for understanding individual coronal alignment variations beyond the broader CPAK categories, potentially revealing more subtle kinematic consequences of alignment changes [11]. Nevertheless, the lack of kinematic differences suggests that factors such as component design, ligament balancing, and implant positioning, facilitated by robotic systems, may drive functional outcomes more than coronal alignment changes. This supports the flexibility of alignment strategies in robotic‐assisted TKA, allowing surgeons to prioritize soft tissue balance without strictly maintaining preoperative CPAK types.

This study has limitations. First, small and imbalanced cohort carries a risk of Type II error and may not be able to detect subtle differences. Second, the 12‐month follow‐up cannot capture long‐term effects on implant longevity or satisfaction. Thirdly, the study population, from an Asian region, may have unique CPAK distributions compared to Western populations, as noted in studies on Indian and Korean cohorts [23, 25], potentially affecting the generalizability. Finally, the mixing of MA and KA strategies without a fully powered adjusted analysis introduces confounding, as the chosen alignment philosophy directly dictates post‐operative CPAK. Future research should explore long‐term outcomes of CPAK changes, including implant survivorship and PROMs beyond 1 year. Comparative studies of alignment strategies (e.g., MA vs. KA) across diverse populations could clarify optimal approaches. Additionally, investigating kinematics in specific activities or patient subgroups may reveal nuanced effects of alignment changes. Regardless, this study suggests that changes in CPAK classification after robotic‐assisted TKA do not adversely affect in vivo kinematics or short‐term clinical outcomes. With precise surgical techniques and modern implant designs, satisfactory results can be achieved regardless of maintaining preoperative alignment phenotypes, offering surgeons flexibility in alignment strategies.

## CONCLUSION

Preliminary evidence suggests changes in CPAK following robotic‐assisted TKA do not affect in vivo kinematics or short‐term outcomes, supporting alignment flexibility when precise techniques are used.

## AUTHOR CONTRIBUTIONS

**Michael Tim‐Yun Ong**: Conceptualization; investigation; methodology. **Patrick Shu‐Hang Yung**: Conceptualization; project administration; supervision. **Cham‐Kit Wong**: Project administration; investigation; data curation; validation; writing—original draft; writing—review and editing. **Richard D. Komistek**: Project administration; validation; visualization. **Kevin Ki‐Wai Ho**: Project administration; formal analysis. **Michael LaCour**: Investigation; methodology; data curation; formal analysis; writing—original draft, writing—review and editing. **Rex Wang‐Fung Mak**: Investigation; methodology; data curation; validation; visualization. **Jonathan Patrick Ng**: Investigation. **Tsz‐Lung Choi**: Investigation; methodology. **Garett Dessinger**: Resources; software; validation; formal analysis. **Gloria Yan‐Ting Lam**: Validation; visualization. **Mingde Cao**: Writing—review and editing.

## FUNDING INFORMATION

The authors have no funding to report.

## CONFLICT OF INTEREST STATEMENT

Michael LaCour reports research support from Zimmer‐Biomet, Smith & Nephew, DePuy Synthes and Total Joint Orthopedics as a principal investigator. Garett Dessinger is a paid employee of Orthopaedic Innovation Technology Center. Richard D. Komistek reports receiving royalties from DePuy Synthes and Smith & Nephew. Michael Tim‐Yun Ong serves as Chief Censor of The Hong Kong College of Orthopaedic Surgeons, Honorary Treasurer of The Hong Kong Orthopaedic Association and board member of the Asian Shoulder Elbow Association (ASEA), Arthroplasty Society in Asia (ASIA) and Asia‐Pacific Knee, Arthroscopy and Sports Medicine Society (APKASS). The other authors declare no conflicts of interest.

## ETHICS STATEMENT

Ethical approval was granted by the local research ethics committee (Approval No.: 2022.637). Written informed consent was received from all participants.

## Supporting information

Figure S1.

Figure S2.

## Data Availability

The data that support the findings of this study are available from the corresponding author upon reasonable request.
