# Supplementary material for: Coronal plane alignment changes do not affect in vivo kinematics for robotically performed total knee arthroplasty
Source: J Exp Orthop. 2026 May 26;13(2):e70776. doi: 10.1002/jeo2.70776 (PMC13240438; doi:10.1002/jeo2.70776)
Supplement: Supplementary file 1 — Figure S1. [file JEO2-13-e70776-s001.docx]

**Supplement Figure 1.** CPAK transition patterns following TKA.

(A) Sankey diagram illustrating transitions between preoperative and postoperative CPAK types. The width of each flow represents the number of patients undergoing a specific transition.

(B) CPAK transition matrix showing the frequency of conversion from each preoperative type (rows) to each postoperative type (columns). Cell values indicate the number of patients, with darker shading representing higher frequencies.
