# Supplementary material for: Coronal plane alignment changes do not affect in vivo kinematics for robotically performed total knee arthroplasty
Source: J Exp Orthop. 2026 May 26;13(2):e70776. doi: 10.1002/jeo2.70776 (PMC13240438; doi:10.1002/jeo2.70776)
Supplement: Supplementary file 2 — Figure S2. [file JEO2-13-e70776-s002.docx]

**Supplement Figure 2. a**HKA and JLO angle pre- and postoperatively.

Scatter dot plots illustrating the distribution before and after TKA across CPAK no change and change group. Each dot represents an individual patient. *** P<0.001.
